# Shaping the Silhouette: A Comprehensive Review of Surgical Body Contouring Techniques for the Torso and Limbs

**DOI:** 10.7759/cureus.88737

**Published:** 2025-07-25

**Authors:** Pawan Acharya, Abisshek Raj Alagarasan, Mahi Khan, Juan Pablo Zuluaga Blanco, Ahsen Cakir, Francesca Abusada, Mohammad F ALQahtani, Humza F Siddiqui

**Affiliations:** 1 Plastic and Reconstructive Surgery, Charing Cross Hospital, London, GBR; 2 Plastic and Reconstructive Surgery, Clarité Hospital, Madurai, IND; 3 Medicine, Baqai Medical University, Karachi, PAK; 4 Medicine, Universidad El Bosque, Bogotá, COL; 5 Research, SerenaGroup Clinical Research, Cambridge, USA; 6 Medicine, Universidad Peruana de Ciencias Aplicadas, Lima, PER; 7 Pediatrics and Child Health, Imam Abdulrahman Bin Faisal University, Dammam, SAU; 8 General Surgery, Jinnah Postgraduate Medical Centre, Karachi, PAK

**Keywords:** aesthetic abdominoplasty, body contouring surgeries, brachioplasty, breast implant, liposuction, thighplasty

## Abstract

This narrative review examines contemporary surgical body contouring techniques, including abdominoplasty, brachioplasty, liposuction, and fat grafting, as well as breast and gluteal augmentation, thighplasty, calf augmentation, and genital contouring, with a focus on clinical applications, technological advancements, complication profiles, and patient-centered outcomes. Abdominoplasty, including mini, lipoabdominoplasty, and fleur-de-lis variants, consistently restores the abdominal contour and can alleviate functional sequelae, with complication rates lowered by the use of progressive tension sutures and preservation of Scarpa fascia. Brachioplasty and thighplasty in post-bariatric patients improve limb contour and mobility; liposuction-assisted approaches reduce tissue trauma. Energy-assisted devices and circumferential 360° liposuction enhance precision and balance. Autologous fat grafting, ranging from macro- to nano-fat, yields high satisfaction and regenerative benefits; however, fat resorption and rare embolic events warrant a careful technique. Breast augmentation, reduction, and mastopexy achieve high patient satisfaction and improved symmetry; combining augmentation with mastopexy addresses ptosis. Calf and genital contouring refine regional aesthetics with the evolution of implant and fat-grafting methods. Overall satisfaction is high across procedures; complication profiles vary by modality and are mitigated by surgeon expertise and patient selection. Modern body contouring techniques deliver transformative aesthetic, functional, and psychosocial benefits. Continued refinement of technology and techniques, rigorous patient selection including psychological readiness, and long-term outcome studies are essential to further optimize safety and patient-centered care.

## Introduction and background

**Figure 1:**
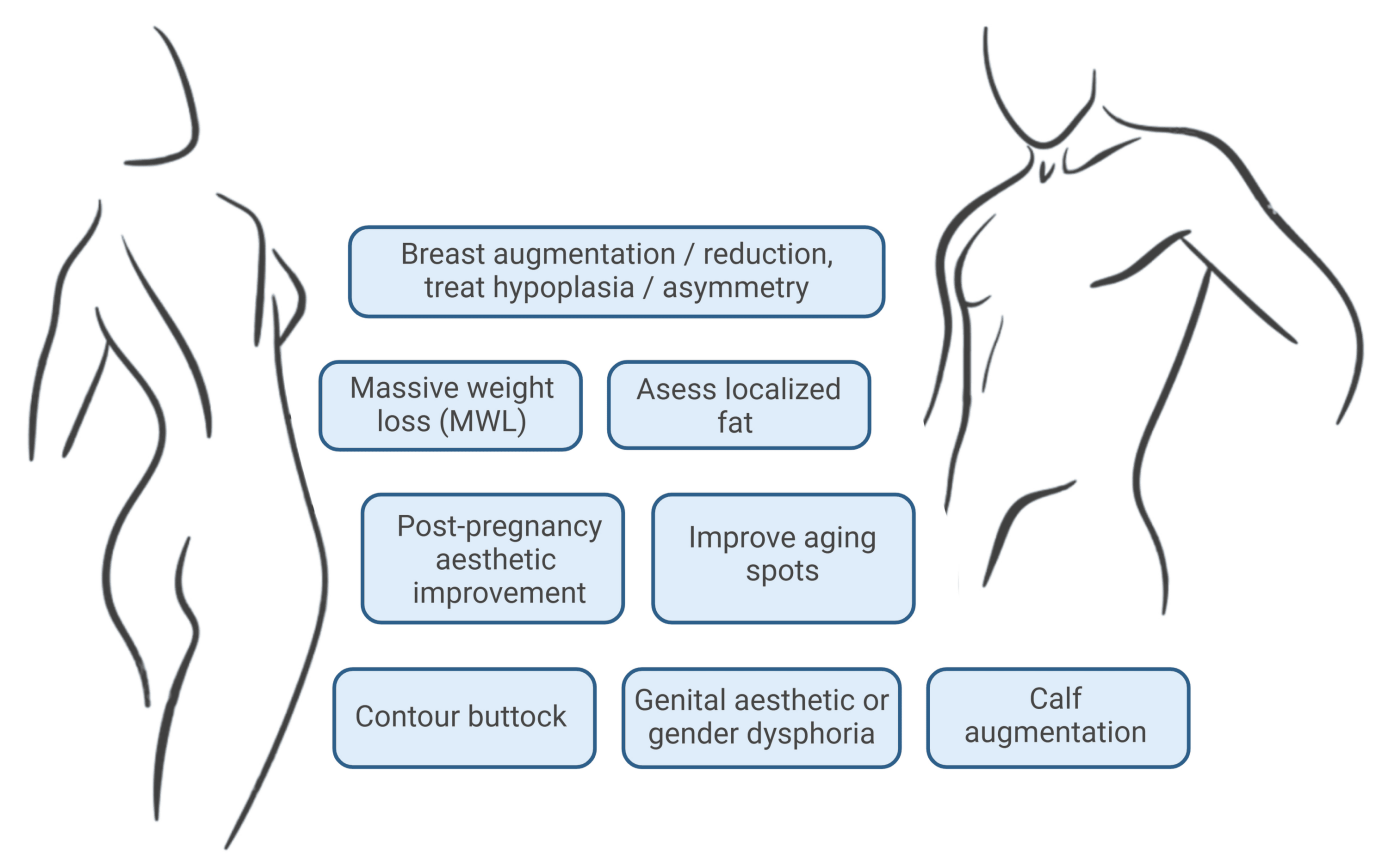
Indications for body contouring procedures Image Credit: Figure made using biorender.com by Juan Pablo

In contemporary society, the significance of physical appearance has become increasingly prominent, driven by factors such as self-esteem and media influence, which have contributed to the rising demand for cosmetic and aesthetic procedures [1]. More recently, there has been a noticeable shift toward a "health-promoting lifestyle" that emphasizes nutritional awareness, regular physical activity, and post-weight loss body reshaping aimed at achieving natural and sustainable aesthetic outcomes. This evolving approach complements traditional interventions for obesity and its sequelae, particularly through body contouring procedures, utilizing both surgical and non-surgical modalities to refine body proportions and enhance physical contours [2]. Body contouring indications have been summarized in Figure 1.

Among the most commonly performed procedures are those involving the breasts. In male patients, surgical treatment typically consists of mastectomy, realignment of the nipple-areola complex (NAC), and chest wall flattening to attain a more traditionally masculine thoracic contour [2,3]. In female patients, breast contouring procedures address dissatisfaction with breast size or shape, contributing to improved body symmetry and enhancing overall aesthetic balance. Abdominoplasty remains a cornerstone technique for improving abdominal profile, targeting excess skin and adipose tissue. Frequently incorporating rectus muscle plication to restore abdominal wall integrity [2,4]. A range of techniques, including mini-abdominoplasty, modified or full traditional abdominoplasty, and lipoabdominoplasty, may be selected based on the patient's specific anatomical and aesthetic needs [5]. Brachioplasty, which addresses redundant tissue in the upper arms, has seen increased utilization, particularly in individuals who have undergone massive weight loss (MWL). Depending on the degree of skin laxity and adiposity, treatment may involve liposuction, direct excision, or a combination of both [6,7].

**Figure 2:**
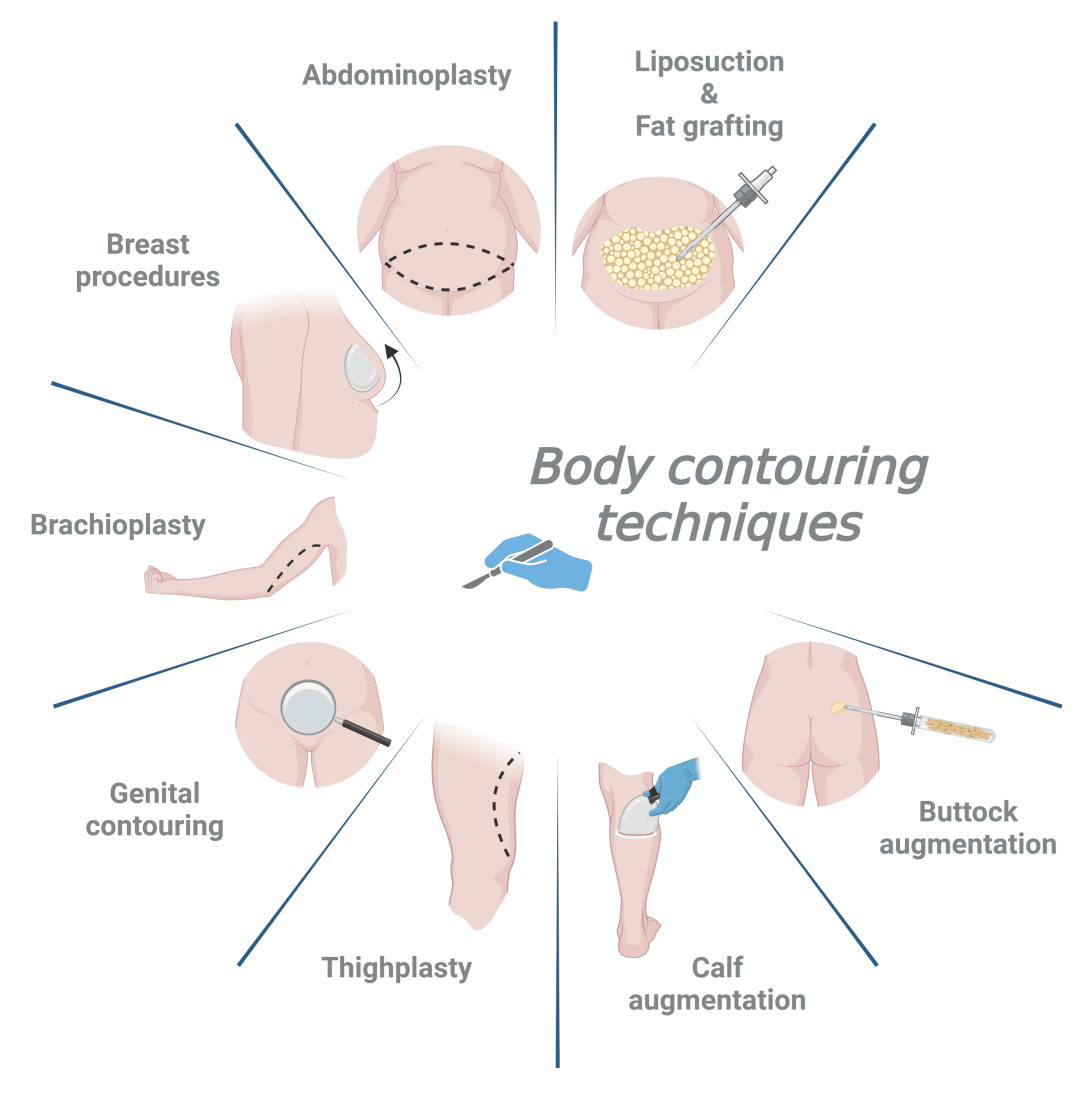
Summary of body contouring procedures Image Credit: Figure made using biorender.com by Juan Pablo and Humza Siddiqui

Various specialized body contouring techniques have been developed to address region-specific deformities and aesthetic concerns. Thighplasty, commonly performed in MWL patients, enhances the lower limb contour through horizontal tension strategies that preserve structural integrity and minimize tissue disruption [8]. Liposuction not only facilitates targeted fat removal but also enables autologous fat transfer to improve contour, symmetry, and definition. Technological advancements, including ultrasonic, laser-assisted, and circumferential approaches, have enhanced the precision and outcomes of these procedures [7,9]. Gluteal augmentation has gained increasing popularity, employing either silicone implants or autologous fat grafting to restore or enhance gluteal volume and shape with natural-appearing results [10]. Similarly, calf augmentation has evolved from the use of solid silicone implants to contemporary techniques that incorporate fat grafting and endoscopic assistance, providing improved definition and reduced morbidity [11]. Genital contouring and rejuvenation procedures encompass both aesthetic refinement and functional restoration. Feminizing procedures, such as clitoroplasty, vaginoplasty, and labiaplasty, aim to enhance genital aesthetics while preserving or improving sexual function [12]. Masculinizing procedures, including metoidioplasty and phalloplasty, serve as critical components of gender-affirming surgical care, providing individualized reconstructive pathways tailored to each patient's anatomy and goals [13]. Additionally, post-weight loss procedures like monsplasty contribute to improved hygiene and a more seamless aesthetic transition between the abdomen and the pubic region [12]. Figure 2 summarizes the body contouring procedures.

This narrative review aims to provide a comprehensive overview of contemporary surgical body contouring techniques for the torso and limbs. It examines their clinical applications, aesthetic and reconstructive outcomes, technical innovations, and challenges in delivering natural, patient-centered results across a diverse population.

Methodology

This structured narrative review was conducted to synthesize current evidence on surgical body contouring techniques, with emphasis on clinical indications, technical innovations, complication profiles, and patient-reported outcomes. A comprehensive literature search was performed using PubMed, MEDLINE, Embase, the Cochrane Library, Scopus, and Google Scholar, targeting English-language articles published between 2010 and 2025. Search terms included combinations of "body contouring", "abdominoplasty", "brachioplasty", "liposuction", "fat grafting", "breast augmentation", "thighplasty", "genital surgery", and "psychosocial outcomes".

Priority was given to high-quality sources, including systematic reviews, randomized controlled trials, meta-analyses, and large cohort studies. Additional references were drawn from expert consensus guidelines and standard surgical textbooks to enhance clinical relevance. Articles were selected based on their applicability to current practice and contribution to understanding aesthetic and functional outcomes. No formal risk-of-bias assessment was conducted, as the review was narrative. Findings were thematically organized by anatomical region and procedural category to provide a comprehensive, evidence-based overview of contemporary body contouring practices.

## Review

Breast procedures in females

**Figure 3:**
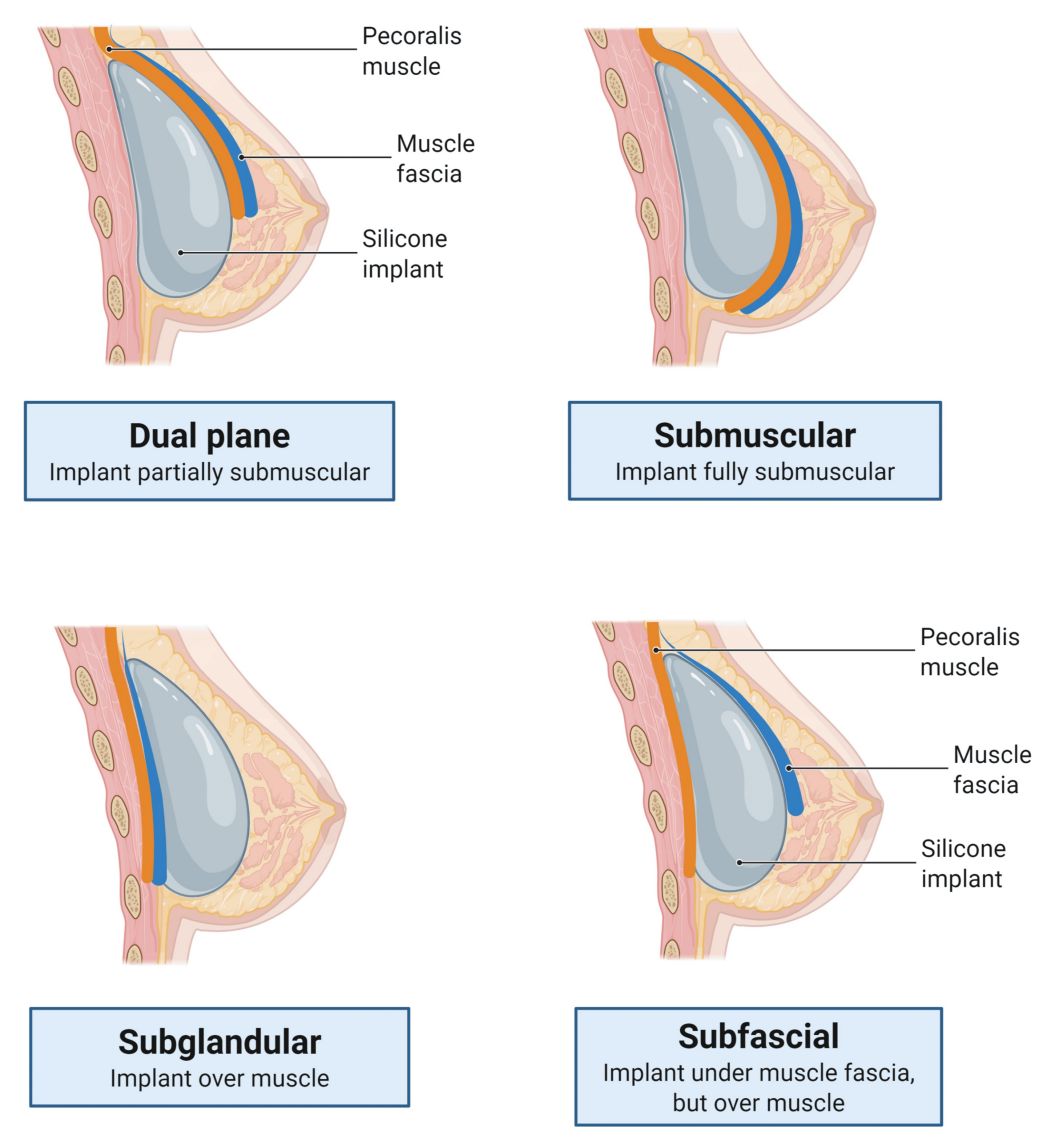
Types of breast implant Image Credit: Figure made using biorender.com by Juan Pablo

Aesthetic breast surgeries such as augmentation, reduction, mastopexy, and fat grafting aim to enhance breast appearance and proportion while improving symmetry. With advancements in surgical methods and implant materials, surgeons are increasingly able to achieve natural results with minimal recovery time. Research in this area explores innovative techniques, implant safety, and the psychological impact of aesthetic breast procedures. The influence of social media has also played a role, as individuals compare themselves to curated images online, which can affect self-esteem and body image, motivating them to pursue surgery [14]. Among the most common procedures are breast augmentation and reduction, often performed to address hypoplastic or hyperplastic breasts. Women experiencing postpartum involution or post-weight-loss volume loss may also seek augmentation, with studies showing that approximately 40% of breast reduction patients report significant weight loss post-surgery and later express interest in volume restoration (p < 0.01 for the impact of postoperative weight reduction on augmentation interest) (Figure 3) [15,16].

Preoperative evaluation is crucial and should involve a detailed medical, surgical, and social history, including comorbidities, bleeding tendencies, smoking, and any familial or personal history of breast cancer. A physical exam should document bilateral measurements and detect any abnormalities in skin or nipple presentation. Essential pre-surgical investigations include a complete blood count and an ECG for patients over 40 years of age. During consultation, setting realistic postoperative expectations is key to patient satisfaction [17]. Aesthetic breast surgery is contraindicated in patients with active infections, untreated breast cancer, autoimmune diseases, hypersensitivity to silicone, or those who are pregnant or breastfeeding. Individuals with mental instability or unrealistic expectations should be discouraged from undergoing surgery [18].

Frequently encountered complications include breast pain, nipple changes, and infections. Risk factors such as elevated BMI, diabetes, smoking, and prior surgeries may contribute to surgical site infections. Postoperative complications include seromas, hematomas, and infections, particularly when drains are used or in the presence of tissue ischemia. Improper use of compression garments or postoperative massage can lead to issues like implant malposition [19,20]. Following breast augmentation, patients often report an improvement in body image and self-esteem, with a mean improvement in body image score of +1.8 (95% CI: 1.2-2.4; p < 0.001). However, some women undergoing mastopexy may experience temporary fatigue or emotional shifts during the early postoperative period, though most report emotional recovery and enhanced well-being within 6 months [21]. Breast reduction surgery has been shown to significantly improve quality of life within three months, often bringing patients to a state of health comparable to that of the general population (p < 0.001; standardized QoL score increase: +23.6 points; 95% CI: 19.4-27.8) [22]. However, the ability to breastfeed may be compromised depending on surgical technique and whether the subareolar parenchyma is preserved. Reasons for not breastfeeding include inadequate milk production and psychosocial concerns [23]. To address the risk of ptosis following augmentation in patients with breast sagging, techniques like augmentation mastopexy are used. This method involves excising the lower pole of the breast to stabilize the tissue and reduce long-term descent, resulting in a recurrence rate of ptosis of 6.8% with augmentation mastopexy compared to 17.4% with mastopexy alone (p = 0.02). Other methods, such as the Benelli (periareolar) mastopexy, may yield less favorable aesthetic results, often leading to tissue flattening and suboptimal contouring [24].

Aesthetic breast surgery in males

Aesthetic breast surgery has gained popularity among men seeking a defined and proportionate chest. This surge in interest reflects broader cultural shifts and heightened awareness of male body aesthetics. Gynecomastia, characterized by benign breast tissue enlargement due to stromal, ductal, or fatty proliferation, remains one of the most frequently performed aesthetic procedures in men [25-27]. Surgical correction typically includes liposuction and gland excision, sometimes paired with fat grafting along the pectoralis margin to enhance contour. Despite variations in technique, the central goal remains consistent: to achieve a masculine chest appearance with minimal complications [28].

Indications include all grades of gynecomastia, lipomastia, and tubular breast deformity. Regardless of cause, treatment generally involves surgical intervention aimed at restoring chest aesthetics. Outdated techniques such as simple mastectomy with free nipple transfer, isolated gland excision, and double incision mastectomy are now avoided due to risks of excessive scarring and distortion of the NAC. Studies have helped define the ideal male chest by assessing surgical outcomes in terms of NAC positioning and chest symmetry, providing valuable reference for both surgeons and patients. Complications such as hematoma and seroma remain concerns; however, the use of advanced techniques, including quilting sutures, fibrin glue, and doxycycline sclerosis, has helped mitigate these risks. Notably, the use of doxycycline sclerosis has led to a significant reduction in the seroma rate, from 14.3% to 4.6% (p = 0.01). Ultrasound-assisted liposuction and power-assisted liposuction, in combination with enhanced tumescent fluid, intraoperative IV tranexamic acid, and careful electrocautery, have proven effective in minimizing bruising, bleeding, and adverse outcomes, with a mean reduction in hematoma volume of 43% (p < 0.05). Postoperative strategies include consistent use of compression garments, lymphatic massage, and NAC lifting plasters, especially beneficial for higher-grade gynecomastia with significant skin laxity and breast ptosis. Preoperative skin quality assessment is also crucial for predicting surgical success and minimizing long-term issues. A significant concern is the rise in aesthetic surgeries performed by unqualified practitioners, both in the U.S. and internationally. Highlighting the need for stringent regulations and patient education to ensure safe and ethical practices within the field of aesthetic breast surgery [25-28].

Abdominoplasty

Abdominoplasty is a cosmetic surgical procedure targeting the abdominal area, involving a horizontal incision in the lower abdomen, elevation of the abdominal flap, correction of rectus diastasis through rectus sheath plication, excision of excess tissue, and closure with umbilical repositioning [29]. This procedure removes redundant abdominal skin and adipose tissue while reinforcing the rectus abdominis musculature [30]. The operation can be performed using various techniques, including conventional abdominoplasty, lipoabdominoplasty, mini-abdominoplasty, circumferential abdominoplasty, extended abdominoplasty, reverse abdominoplasty, and fleur-de-lis/inverted-T excision [31].

Abdominoplasty has gained popularity for aesthetic and functional improvement, especially among women with abdominal wall laxity post-pregnancy, bariatric patients following a significant weight loss with excessive skin and/or pannus, and older adults who intend to maintain a youthful physique. Additionally, the wide abdominal rectus plication abdominoplasty procedure has also gained attention for its therapeutic potential in addressing conditions such as back pain, urinary incontinence, and neuro-myodystrophy [30]. Although considered safe, abdominoplasty carries a complication rate of approximately 2.1% [31], which is notably higher than the 1.4% complication rate observed in other aesthetic surgeries (p = 0.04; 95% CI: 0.006-0.034) [32]. A significant variation in complication rates exists among different types of abdominoplasties. Among the seven types studied, fleur-de-lis abdominoplasty has the highest overall complication rate, reaching 3.81% (p < 0.001 when compared with mini abdominoplasty; 95% CI: 1.9-6.4) [31].

Common complications include seroma, wound infections, hematoma, flap and/or fat necrosis, and thromboembolism. Out of all the complications, seroma is the most common, affecting five to 30% of patients [30]. According to Salari et al., the prevalence of seroma post-classical abdominoplasty is 10.9% [33]. A study by Bromley et al. showed rates as high as 76.2% [34]. Seroma refers to a collection of fluid in the dead space between the fascia and muscle, which often necessitates repeated aspiration, thereby increasing the risk of infection and prolonged recovery and occasionally requiring additional surgical treatment [30]. Techniques such as progressive tension sutures (PTS) (OR: 0.36, 95% CI: 0.19-0.70; p = 0.003), PTS with liposuction (OR: 0.24, 95% CI: 0.11-0.49; p = 0.0001), and Scarpa fascia preservation (OR: 0.229, 95% CI: 0.13-0.40; p < 0.001) significantly reduce seroma risk [34,35]. A meta-analysis comparing general techniques to conventional drainage methods found a significantly lower risk of complications with general techniques, with a risk ratio of 0.33 (95% CI: 0.11-0.99; p = 0.045). Despite this, Seretis et al. recommend the use of closed suction drains as the standard of care for seroma prevention, even though these drains are associated with increased postoperative pain and complications such as reverse migration and bacterial contamination [36]. Proper case selection and risk-stratified prophylaxis of thromboembolism are essential in effectively reducing the occurrence of other postoperative complications following abdominoplasty [34,35].

Brachioplasty

Brachioplasty, also known as an upper arm lift, removes excess skin and fat from the upper arms, improving both contour and function. It helps address issues like intertrigo, poor hygiene infections, and psychosocial difficulties that may arise from excess skin or ptosis. This procedure is especially beneficial for patients who have experienced significant weight loss, such as after bariatric surgery, as it promotes contentment and a better quality of life, resulting in a reduced BMI. Assessment of the patient's skin and fat is essential for selecting the best procedure, which may include brachioplasty alone or combined with liposuction [37].

Brachioplasty is typically performed on patients with significant weight loss, aging, or those who have experienced pregnancy. There are various techniques for arm contouring, such as liposuction (e.g., suction-assisted or power-assisted), cryolipolysis, and radiofrequency treatments, which can be alternatives to brachioplasty. For optimal results, patients should maintain a stable weight for three to six months after bariatric surgery, ideally for 12 months or longer. Contraindications include poor general health, active tobacco use, and unrealistic expectations regarding surgical outcomes. Additionally, individuals with conditions like extensive lymphadenectomy, lymphedema, or compromised vascular function should avoid it [37,38]. Postoperative complications can range from minor issues, such as wound infection or seroma, to more serious concerns like bleeding or thromboembolism. Smokers are at a higher risk of wound healing problems, including infections and hematomas. Tobacco use impairs wound healing by causing vasoconstriction and microvascular injury, which hinders oxygen supply and increases the likelihood of complications. Comorbid conditions like hypertension, obesity, and diabetes are common in patients undergoing body contouring surgeries and can influence the outcome [39,40]. To minimize complications, techniques such as the "enhanced avulsion technique" and the "jaws" technique are used. The enhanced avulsion technique provides a thin flap with minimal subcutaneous tissue, preserving neurovascular structures, and has been reported to significantly reduce seroma and hematoma rates (p < 0.01). It also reduces the need for drain placement, enabling ambulatory care and resulting in cost savings. The "jaws" technique utilizes dermal flaps to minimize scarring and tension at the surgical site, resulting in improved cosmetic outcomes. These methods aim to enhance healing and minimize complications (p = 0.02) [41,42].

The impact of brachioplasty on body image is significant, especially for women, as it can improve the perceived attractiveness of the upper arms. Slim, toned arms are often perceived as more feminine, and the procedure plays a significant role in enhancing social and nonverbal communication. While patient-reported outcomes are generally positive, patients' expectations for body contouring surgeries can sometimes be unrealistic, and surgeons may struggle to meet these high expectations [43].

Buttock augmentation

The growing demand for buttock augmentation has spurred advancements in surgical techniques, particularly autologous fat grafting, which offers natural results with a lower risk of rejection compared to synthetic implants. Studies have highlighted the effectiveness of ultrasound-assisted liposuction and ultrasound-guided fat grafting, with a 78% patient satisfaction rate reported six months post-procedure in 185 patients. These methods improve fat harvesting precision and minimize complications, such as fat necrosis and embolism (p < 0.01, 95% CI: 0.08-0.32) [44].

Autologous fat grafting is typically indicated for patients seeking to enhance their buttock volume and contour, often due to aging, weight loss, or genetic factors. Ideal candidates have a body fat percentage of 20-30% and a BMI between 20 and 30 kg/m². This method is favored by those who prefer natural enhancements over implants and by individuals who have previously undergone liposuction elsewhere [45,46]. However, contraindications must be considered, such as significant comorbidities, an unsuitable BMI, smoking, or unrealistic expectations. These factors can increase the risk of complications or lead to dissatisfaction [44-46]. Favorable outcomes include improved body contour, enhanced self-esteem, and high patient satisfaction, with 78% of patients expressing satisfaction six months after the procedure. This technique has proven beneficial for individuals seeking to enhance their body proportions and boost confidence [44].

Despite its benefits, buttock augmentation carries risks like fat necrosis, seroma formation, infections, and asymmetry. Complication rates vary, with fat grafting reported to have a complication rate of 10.5%. Fat embolism, though rare, is a serious concern, occurring in 1-3% of cases, with some fatalities. Seromas and infections can also occur, but are generally manageable. Achieving symmetry can be challenging, as fat resorption affects long-term results, necessitating additional procedures in 30-50% of cases [45,47]. Patient satisfaction remains high, especially with autologous fat grafting. A study reported 78% satisfaction six months post-surgery, with favorable results associated with a healthy BMI and proper counseling [44]. Advancements such as ultrasound guidance for fat placement and improved injection techniques have enhanced outcomes. Preoperative imaging technologies and postoperative care protocols also contribute to a faster recovery and fewer complications [46,48].

Thighplasty

Thighplasty, also known as thigh lift surgery, is a cosmetic and reconstructive procedure that removes excess skin and fat, reshaping the thighs for a more youthful and balanced appearance [49]. Since its inception in the 1950s, the procedure has undergone significant evolution, enhancing both safety and outcomes [50]. Early techniques often led to complications and visible scarring, but the integration of liposuction and tissue-preserving methods has improved results and patient satisfaction [51]. This review outlines the indications, contraindications, surgical approaches, and advancements in thighplasty. Thighplasty is performed on patients with excess skin following MWL, often after bariatric surgery. It is also beneficial for addressing age-related skin sagging, which can cause discomfort and aesthetic concerns [52]. Medial thighplasty removes excess skin from the inner thigh through a groin incision [53], while lateral thighplasty targets the outer thigh and hip area. Circumferential thighplasty addresses the removal of extensive skin and fat, typically in patients who have undergone significant weight loss [54]. Thigh lifts can lead to improved mobility, a better appearance, and an enhanced quality of life, with minimal scarring when performed correctly [55].

However, patients with medical conditions like uncontrolled diabetes, bleeding disorders, or infections have increased surgical risks [56]. Poor skin elasticity, resulting from factors such as aging, smoking, or sun damage, limits aesthetic outcomes [57,58]. Other contraindications include venous insufficiency, lymphedema, or a tendency for hypertrophic or keloid scarring [59]. A thorough preoperative assessment is essential to determine candidacy. Thighplasty generally improves the contour of the thighs and body proportion. Patients also report increased confidence and comfort in daily movement after removing excess skin [60]. Additionally, it relieves skin irritation and discomfort caused by redundant folds [61,62]. Complications may include wound dehiscence, seroma formation, infection, scarring, or asymmetry [60]. These risks can be mitigated through surgical techniques, effective postoperative wound care, and comprehensive patient education. In some cases, follow-up surgeries may be necessary to address issues such as uneven contour or poor scarring.

Recent advancements have made the procedure safer and more effective. Liposuction-assisted thighplasty allows for more precise contouring and fewer complications compared to traditional methods (p = 0.014, 95% CI: 0.05-0.42) [63]. Energy-based devices, such as radiofrequency and ultrasound, support skin tightening and tissue reshaping [64]. Meanwhile, 3D imaging tools aid surgeons in planning more accurate and personalized procedures [65]. Combining thighplasty with other body contouring surgeries, such as abdominoplasty or brachioplasty, has become increasingly popular for comprehensive results [66]. Recovery protocols using modern pain management and suture techniques further improve healing and patient satisfaction [67]. Thighplasty remains a valuable option for improving body contour and function, particularly after significant weight loss. Advances in technique and technology continue to enhance outcomes and reduce risks. Future research should investigate long-term outcomes to understand their impact on patient well-being better.

Calf augmentation

Calf augmentation is a growing aesthetic procedure for both men and women seeking fuller, more defined legs. Thin calves, whether due to genetic predisposition, trauma, or muscle deficiency, are often considered aesthetically undesirable, and therefore, the use of alloplastic prosthesis is becoming more accepted and increasingly requested worldwide.

The procedure enhances leg contour and restores balance between the thighs and calves, particularly among bodybuilders. Calves are the focal point of aesthetic assessment of the legs. Calf shape is mainly determined by the development of the soleus and gastrocnemius muscles, the length and orientation of the lower leg bones, and the distribution of the subcutaneous fat [68]. Techniques include non-surgical options, such as hyaluronic acid and PMMA fillers, as well as surgical methods, including silicone implants and autologous fat grafting. The principle of augmenting calves with implants made of solid silicone rubber was first introduced by Carlsen in 1972 and subsequently independently published by Carlsen and Glitzenstein in 1979 [68,69]. Silicone implants can be placed in either the subfascial or submuscular plane, with the latter offering lower complication rates (0.92%) and non-palpability of the implant but more postoperative pain. The submuscular plane is associated with a significantly lower complication rate (0.92% vs. 5.7% in subfascial placement; p = 0.008, 95% CI: 0.01-0.48) and better implant concealment, although it results in greater postoperative discomfort [68]. Subfascial placement remains more common due to ease and reliable cosmetic results, though seroma formation is more frequent. Autologous fat grafting is a less invasive alternative for congenital calf asymmetries and pseudo-varus deformity. It offers smaller incisions, simultaneous liposuction, and fewer foreign body reactions but often requires multiple touch-up sessions due to inconsistent volume retention [70].

Genitoplasty

Concerns about penile size can negatively affect men’s self-esteem and mental health, often leading to anxiety and depression [71]. Dissatisfaction with genital dimensions drives interest in both surgical and non-surgical augmentation [72], with approximately 10,000 cosmetic penile procedures performed in the U.S. over the past decade [73]. Surgical interventions are primarily indicated for anatomical abnormalities such as Peyronie’s disease or congenital micropenis (>2.5 SD below the mean) [72]. Lengthening techniques include suspensory ligament release, plasties, and phalloplasty. Girth enhancement involves fillers, grafts, scaffolds, and Penuma [72]. In one prospective cohort, suspensory ligament release combined with silicone sheath stabilization showed significantly reduced reattachment rates (p = 0.038) and improved patient satisfaction scores (mean change = +1.6 on the IIEF scale, 95% CI: 0.4-2.8) [73,74]. Suspensory ligament release may result in reattachment and altered erectile angles; stabilization using fat flaps or silicone sheaths can mitigate these issues [73]. Current evidence is limited by methodological flaws, underscoring the need for standardized research [72].

In women, aesthetic genital surgery is often pursued to modify labial appearance [75]. Procedures such as labiaplasty and autologous fat grafting also address functional issues, including vulvovaginal laxity and atrophic vaginitis, by leveraging adipose-derived stem cells for tissue regeneration [76]. Despite general safety, risks include infection, fat necrosis, and asymmetry [75].

Liposuction and liposculpture

**Figure 4:**
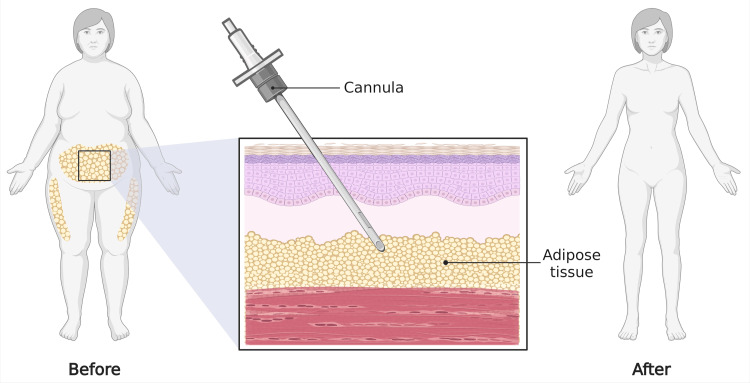
Liposuction Image Credit: Figure made using biorender.com by Juan Pablo and Humza Siddiqui

Liposuction is a well-established cosmetic procedure that utilizes suction-assisted cannula insertion to remove localized fat deposits. Techniques have advanced from the traditional “dry” method to more refined approaches, such as tumescent, ultrasound-assisted, and laser-assisted liposuction, which enhance contour precision and reduce complications (Figure 4) [77].

It is primarily indicated for patients with localized fat deposits resistant to diet and exercise, commonly affecting the abdomen, thighs, arms, or chin. Candidates should have good skin and muscle tone. Beyond aesthetics, liposuction is also used to treat conditions such as obesity-related metabolic syndrome, lower limb arthritis, lipedema, gynecomastia, HIV-induced lipodystrophy, lymphedema, and other disorders affecting fat distribution [78]. Contraindications include poor skin elasticity, significant comorbidities such as uncontrolled diabetes and coagulopathies, unrealistic expectations, and diffuse obesity, where bariatric surgery is preferred. It is often combined with procedures like abdominoplasty in post-bariatric patients [79]. When used appropriately, liposuction yields notable improvements in contour through small incisions, resulting in high patient satisfaction. Advances in anesthesia and fluid management have reduced recovery time and improved safety. Minimal scarring and downtime also contribute to favorable psychological and aesthetic outcomes [80].

However, complications include contour irregularities, seroma, hematoma, infection, fat or pulmonary embolism, volume overload, panniculitis, and rare conditions like sepsis or gas gangrene. Risks are minimized through patient selection, adherence to technique, fluid and temperature management, and postoperative care that includes the use of compression garments and lymphatic massage [81]. Recent innovations include energy-based devices (e.g., ultrasound, laser, radiofrequency, and power-assisted liposuction), which enable better fat emulsification and removal with reduced tissue trauma. While water- and laser-assisted methods are available, they carry higher complication rates and are less commonly used [82,83].

Three hundred and sixty-degree liposuction, also known as circumferential liposuction, targets fat around an entire area (typically the torso) for a balanced, hourglass-like result. It is suitable for individuals with significant circumferential fat or those who have undergone MWL. Contraindications include poor skin elasticity, comorbidities, and a high risk of complications related to anesthesia. Despite concerns, studies have shown comparable complication rates between 360-lipo-abdominoplasty and traditional abdominoplasty in high-risk patients (p = 0.12) [84,85]. Complications may include prolonged edema, asymmetry, and contour irregularities, particularly if fat removal is uneven or incomplete. Arbitrary lipoaspirate limits (e.g., 500-1000 ml) per area lack strong evidence for risk reduction [86]. When performed skillfully, this approach enhances symmetry, waist-to-hip ratios, and patient satisfaction, especially when combined with thorough counseling and expectation setting [87,88]. Fat transfer, also known as liposculpture, involves harvesting, processing, and reinjecting fat for contour enhancement or volume restoration. Depending on particle size, it is classified into macro, micro, nano fat, or stromal vascular fraction. This method provides natural, biocompatible augmentation and has demonstrated benefits in tissue regeneration, scar improvement, radiation damage, chronic wound healing, and vocal cord repair due to its high content of mesenchymal stem cells [89,90].

Refined fat grafting techniques: macro, micro, and nano fat

Macrofat grafts, composed of adipose particles larger than 2.4 mm, are typically used for structural augmentation in high-volume areas such as the breasts and buttocks. These are injected with blunt needles (≥2 mm) and are ideal for volumetric enhancement in the breast, abdomen, hands, and calves. Numerous successful breast lipofilling techniques have been reported globally [91,92]. Contraindications include poor vascularity or active infection. When properly harvested and processed, macro fat, along with micro and nano fat, can volumize and rejuvenate tissues while maintaining a natural look. However, complications may occur, such as oil cysts, palpable masses, seroma, infections, fat embolism, DVT, pulmonary or cardiac events, and rare cases of breast cancer recurrence. Overcorrection, resorption, and localized issues, such as swelling or nodularity, have also been noted, with complication rates varying by treatment site [93]. Patient satisfaction remains high when natural contours are achieved.

Microfat grafting utilizes more refined fat particles (≤ 1.2 mm) and is well-suited for delicate areas, such as the periorbital region, hands, nose, and forehead. It is typically harvested using cannulas and emulsifiers with openings of 1.2-2.4 mm [94]. Best used for subtle enhancements and contour correction, microfat is especially valuable in facial bone hypoplasia, offering a less invasive alternative to surgical osteotomies [95]. Contraindications are similar to those for macro fat, particularly in scarred or poorly vascularized tissue. It provides better tissue integration with fewer visible irregularities, though uneven distribution may still result in aesthetic shortcomings. Satisfaction is generally high when natural enhancement is the goal.

Nanofat is a highly refined emulsion rich in stem cells and growth factors, derived through further processing of harvested fat. Typically, it is extracted using 1.2-2.4 mm cannulas and emulsifiers with apertures of 400-600 µm [96]. Unlike macro- and microfat, nanofat is not used for volume restoration but for skin rejuvenation, treating superficial wrinkles, scars, chronic wounds, and overall tissue quality improvement (p < 0.01; 95% CI: 0.72-0.94) [97]. It is often used in conjunction with microfat, where the latter adds volume and the former enhances skin health. Contraindications include active infections or unrealistic expectations of volumetric results. While outcomes include enhanced skin texture and reduced scarring, retention is unpredictable, and repeat treatments may be required. Still, patient satisfaction remains high due to the regenerative benefits that the procedure offers. Advances in emulsification, centrifugation, and filtration techniques have improved the efficacy and minimized cell trauma during processing [98]. The progression of liposuction techniques and fat transfer from macro to nano fat demonstrates a growing refinement in body contouring and rejuvenation. Each method has distinct applications and outcomes. Technological and procedural innovations have contributed to increased safety and long-term results. Ongoing research is expected to further optimize these procedures.

Discussion

In recent years, the field of body contouring has undergone considerable evolution, offering a range of surgical techniques tailored to meet individual anatomical and aesthetic objectives. Procedures such as abdominoplasty, brachioplasty, liposuction, fat grafting, breast and gluteal augmentation, thighplasty, and genital surgeries are now commonly performed to address both post-weight loss deformities and aesthetic enhancement. While each technique presents distinct advantages, they also involve specific risks and must be carefully matched to patient profiles through thorough preoperative assessment.

One of the most consistently reported benefits of body contouring procedures is the significant improvement in patients' overall quality of life, self-esteem, and body image. For instance, autologous fat grafting in buttock augmentation has demonstrated a 78% patient satisfaction rate at six months postoperatively (p < 0.01, 95% CI: 0.08-0.32) [44]. Functional and psychological benefits have also been well-documented following procedures such as brachioplasty and thighplasty, particularly among post-bariatric patients with redundant skin and altered body image perception [37,55]. The introduction of refined liposuction and fat grafting techniques, including macrofat, microfat, and nanofat applications, has enhanced outcomes. These approaches not only offer improved contouring capabilities but also contribute to tissue regeneration and reduced postoperative morbidity (p < 0.01; 95% CI: 0.72-0.94) [89,90,97]. Minimally invasive, these innovations have enabled faster recovery, reduced scarring, and high patient satisfaction.

Despite these advancements, surgical complications remain an inherent consideration. Fleur-de-lis abdominoplasty is associated with the highest overall complication rate (3.81%) among body contouring techniques, mainly due to the extensive tissue manipulation and prolonged operative time [31]. Conversely, submuscular calf augmentation has shown a notably low complication rate of 0.92%, with favorable aesthetic outcomes and limited postoperative morbidity (p = 0.008, 95% CI: 0.01-0.48) [68]. Seroma formation is one of the most prevalent complications following abdominoplasty, with reported incidence rates ranging from 10.9% to as high as 76.2% [34]. In fat grafting procedures, complications such as fat necrosis, asymmetry, and, rarely, fat embolism, particularly in gluteal augmentation (1-3%), require careful technique and patient selection [45]. Surgical outcomes are linked to the experience and technical expertise of the operating surgeon. Techniques such as PTS, preservation of the Scarpa fascia, ultrasound-guided fat injection, and enhanced avulsion methods have been shown to reduce complications when applied appropriately by skilled practitioners (p = 0.0001, 95% CI: 0.11-0.49) [34,41,46]. Conversely, procedures performed by inadequately trained or unqualified providers are associated with significantly higher complication rates, underscoring the need for rigorous surgical training and patient awareness [28].

Psychological considerations in body contouring

Body contouring procedures have a profound impact on patients’ psychological well-being, often resulting in enhanced self-esteem, increased confidence, and an overall improvement in quality of life. However, these benefits are not universal. Individuals with unrealistic aesthetic expectations or underlying psychiatric conditions, particularly body dysmorphic disorder (BDD), are at heightened risk for postoperative dissatisfaction. BDD, which affects approximately 7-15% of patients seeking cosmetic surgery, is characterized by a preoccupation with perceived physical flaws and frequently leads to repetitive surgical interventions with little to no psychological benefit. Preoperative identification of such patients is critical and can be facilitated through validated tools such as the Body Dysmorphic Disorder Questionnaire and structured psychological interviews. Integrating psychological assessment into the surgical evaluation process, preferably in collaboration with mental health professionals, supports ethical patient selection, reduces the likelihood of postoperative regret, and promotes sustained psychological well-being.

All body contouring techniques are summarized in Table 1.

**Table 1:** Overview of body contouring procedures MWL: massive weight loss, DVT: deep venous thrombosis, UAL: ultrasound-assisted liposuction, PAL: power-assisted liposuction, PTS: progressive tension sutures

Procedure	Indication	Common complications	Satisfaction rate	Notable innovation
Abdominoplasty [30-36]	Post-pregnancy, MWL	Seroma (up to 76.2%), DVT	High	PTS
Brachioplasty [37-43]	MWL, aging	Scar, infection	High (especially in women)	Enhanced avulsion technique
Liposuction [77-90]	Localized fat, contouring	Hematoma, embolism	High	Energy-assisted devices (UAL, PAL)
Buttock augmentation [44-48]	Aesthetic purposes	Fat necrosis, fat embolism (1–3%)	High	Ultrasound-guided injection
Breast augmentation [14-24]	Hypoplasia, asymmetry	Infection, implant ptosis	High	Augmentation-mastopexy combo
Male breast reduction [25-28]	Excess gland/fat tissue (gynecomastia)	Seroma, hematoma	High	UAL + NAC lifting
Thighplasty [49-67]	Post-weight loss skin sagging	Wound dehiscence, asymmetry	High	Liposuction-assisted resection
Calf augmentation [68-70]	Thin calves, trauma correction	Seroma (subfascial > submuscular)	High	Endoscopic + fat graft hybrid
Genital contouring [71-75]	Aesthetic or gender dysphoria	Scarring, infection, asymmetry	Variable	Autologous fat for rejuvenation

## Conclusions

Body contouring techniques offer transformative physical and emotional benefits, but they must be performed with precision, supported by evidence-based practices, and tailored to each patient's individual needs. Advancements in technology and technique continue to reduce complication rates and improve satisfaction, but the surgeon's judgment and the patient's psychological readiness remain equally vital to a successful outcome. Future research should focus on long-term outcomes, integration of psychological support, and refining surgical protocols to enhance safety and patient-centered care.
